# Duloxetine reduces pain after Total hip arthroplasty: a prospective, randomized controlled study

**DOI:** 10.1186/s12891-021-04377-4

**Published:** 2021-05-28

**Authors:** Hao Li, Wei-Nan Zeng, Zi-Chuan Ding, Ming-Cheng Yuan, Yong-Rui Cai, Zong-Ke Zhou

**Affiliations:** 1 Department of Orthopaedics, West China Hospital, Sichuan University, 37# WuhouGuoxue Road, Chengdu, China; 2 Department of Orthopaedics, Chongqing General Hospital, University of Chinese Academy of Sciences, Chongqing, China

**Keywords:** Duloxetine, Total hip arthroplasty, Postoperative pain

## Abstract

**Background:**

Previous studies have demonstrated the efficacy of duloxetine in reducing postoperative pain and opioid consumption. However, the effect of duloxetine on total hip arthroplasty (THA) remains unclear. The objective of this study was to assess the efficacy of oral duloxetine in THA.

**Methods:**

We enrolled 96 patients in this randomized controlled trial. These patients were randomized (1,1) to either the duloxetine group or the placebo group and received daily doses of 60 mg duloxetine or placebo, respectively, from 2 d pre-operation to 14 d after surgery. The primary outcome was pain severity upon movement measured by a visual analogue scale (VAS). The secondary outcomes included VAS scores for resting pain, morphine consumption, Harris Hip Score, patient satisfaction at discharge, length of postoperative hospital stay, and adverse events.

**Results:**

Patients in the duloxetine group had significantly lower pain severity scores upon movement within 3 postoperative weeks (*p* < 0.05) while none of the differences met the minimum clinically important difference (MCID). Moreover, patients in the duloxetine group performed better in terms of resting pain (in 3 weeks after surgery), morphine requirements, and satisfaction level at discharge (all p < 0.05). There was no difference between groups in the prevalence of adverse events.

**Conclusions:**

Although it did not result in a clinically meaning reduction in pain after total hip arthroplasty, perioperative administration of 60 mg of duloxetine daily significantly alleviated pain in the postoperative 3 weeks and morphine requirements during the postoperative 48 h. Therefore, duloxetine still shows promise in optimizing the multimodal pain-management protocols in total hip arthroplasty.

**Trial registration:**

Chinese Clinical Trial Registry, ChiCTR2000033606, 06/06/2020.

## Background

Total hip arthroplasty (THA) is a common surgical procedure used to successfully, economically, and safely treat end-stage joint diseases that may cause deformities, lower the quality of life, and lead to bodily dysfunction [1]. Despite excellent outcomes, the overall incidence of dissatisfaction associated with THA is relatively high, at approximately 20% [2].

Approximately 7 to 23% of THA patients suffer postoperative pain, which is one of the most significant unfavorable outcomes related to this procedure [3, 4]. Postoperative pain seriously affects perioperative mood, interferes with joint function recovery, prolongs hospitalization, increases medical expenses, and further reduces the quality of life and work [5]. Inadequate perioperative pain management is associated with a distinct possibility of suffering postoperative pain and numerous other serious complications such as joint stiffness, deep vein thrombosis, and pulmonary embolism [5–8].

Severe postoperative pain exacerbates opioid usage and subsequent opioid-related deaths [6]. In 2017, death rates related to overdosing on opioids, including methadone and heroin, rose sharply to 19.3 per 100,000 in the United States [9]. Opioid prescriptions dispensed by orthopedic doctors accounted for a considerable percentage (7.7%) of prescriptions [10]. Various multimodal analgesia regimens have been proposed to alleviate postoperative pain and reduce side effects during the use of postoperative opiates. Further optimization of perioperative pain management has always been a major topic of related research efforts [6, 7].

Duloxetine (Cymbalta) is a selective serotonin and norepinephrine reuptake inhibitor (SNRI), approved by the Food and Drug Administration (FDA) for the treatment of depression, generalized anxiety disorder, and chronic musculoskeletal pain (osteoarthritis, fibromuscular pain, and chronic back pain, among others) [11, 12]. Duloxetine, which promotes the downregulation of inhibitory pain pathways in the central nervous system, effectively ameliorates pain associated with hyperexcitability corresponding to peripheral sensitization, as well as central sensitization (CS), caused by chronic joint pain [12, 13].

However, as far as we know, all previous studies investigating the effects of duloxetine on pain following arthroplasty are focused on total knee arthroplasty (TKA) [12, 14, 15]. To the best of our knowledge, none of the studies have investigated whether duloxetine alleviates postoperative pain following THA. Based on the hypothesis that duloxetine significantly relieves postoperative pain following THA and leads to further positive postoperative outcomes, we conducted a randomized, double-blinded, placebo-controlled study to determine whether duloxetine optimizes perioperative analgesia protocols in THA.

## Methods

A prospective study, in the form of a single-center (West China Hospital of Sichuan University), randomized, double-blinded, parallel-arm, placebo-controlled clinical trial, was conducted. In this study, we enrolled 153 patients who were scheduled for THA from June 2020 to September 2020. The study was approved by the Ethics Committee on Biomedical Research, West China Hospital of Sichuan University (approval no. 2020–843) and registered at the Chinese Clinical Trial Registry (ChiCTR2000033606, 06/06/2020). Informed consent and research authorizations were obtained from all participants. The research report met unified clinical trial reporting standards and conformed with the Declaration of Helsinki [16].

Eligible patients who were over 18 years of age; classified under American Society of Anesthesiologists (ASA) status I, II, or III; and scheduled to undergo primary THA for end-stage hip joint diseases, were screened using the Hamilton Depression Scale (HAMD) and the Hamilton Anxiety Scale (HAMA). Subjects whose HAMD and HAMA scores were both < 7 were included. The exclusion criteria were as follows: a known allergy to any of the studied drugs, previous exposure to SNRIs or selective serotonin reuptake inhibitors; known psychiatric disorders; alcohol or opioid abuse; acute infections of the hip joint; recent treatment for malignant diseases; major previous ipsilateral hip arthroplasty or open surgery; peripheral or central nerve impairment; cognitive dysfunction; history of peptic ulcers or bleeding tendency; impaired liver and/or renal function; and poor physical condition indicating the lack of ability to tolerate surgery.

Sample size estimation was determined based on previous studies [12, 17]. A sample size of 48 patients in each group was required to test this 2-tailed hypothesis at a power of 0.80, an alpha level of 0.05, and a dropout rate of 20% for detecting a 2-point difference in the pain severity score between groups following surgery. The pain severity score was evaluated using a visual analog scale (VAS) consisting of a horizontal line divided into 10 equal parts. The ends of the horizontal line were marked “0” and “10” and were used to represent no pain to severe pain, respectively; the middle area represented different degrees of pain. The 2-point difference was determined to be the minimum clinically important difference (MCID) because the average acceptable VAS pain score difference following surgery was approximately 2 points according previous studies [12, 18, 19].

Participants were randomized (1,1) to either the duloxetine group or the placebo group. Randomization was concealed from researchers as well as from the patients, by way of sealed envelopes delivered following hospitalization. A sealed, opaque envelope, containing the randomized grouping plan was prepared in advance. After an eligible patient was assigned a sickbed and excluded from surgical contraindications, an independent researcher opened the randomized envelope in the order in which the patients were enrolled to determine the grouping of that patient. Neither the participants nor the primary investigator was aware of the grouping status until the data analysis stage at the end of the study. The hospital pharmacy prepared two types of indistinguishable capsules containing either 60 mg of duloxetine or a placebo (starch) for daily oral administration starting from 2 d pre-surgeryto 14 d post-surgery. Surgery was performed by the same senior doctor, an experienced surgeon who had performed over 300 THA annually. All participants accepted a multimodal and standardized analgesic strategy. From preoperative day 2 to the day before surgery, every patient was given celecoxib 200 mg twice a day (one dose after breakfast and one dose after dinner) for preemptive analgesia. During the operation, all patients received general anesthesia, which was composed of an induction of sufentanil 0.5 μg/kg, midazolam 0.04 mg/kg, propofol 1–2 mg/kg and cistracurium 2 μg/kg intravenously, and a following continuous intravenous infusion of 0.1–0.3 μg/(kg•min) of remifentanil, 2–5 mg/(kg•h) of propofol and inhalation of sevoflurane to maintain anesthesia. Besides, all patients were treated with an 80 mL periarticular injection of 0.25% ropivacaine for local infiltration analgesia. Since postoperative day 1, every patient restarted oral administration of celecoxib (200 mg twice a day) until 2 weeks after the surgery. When acute pain was unbearable or VAS was > 6, morphine (5 mg intravenously) was used as a rescue analgesic [20, 21]. In the perioperative period, there was no other oral analgesics except celecoxib, and intravenous morphine was the only rescue analgesic before discharge. Patients’ discharge criteria for postoperative pain included: pain must be tolerable without affecting daily life and rehabilitation, and the severity of acute pain no longer required intravenous morphine to relieve. All participants were followed up for 3 months.

The pain severity score upon movement (3 h, 6 h, 12 h, 24 h, 48 h, 72 h, 1w, 3w, and 3 m following surgery) was considered as the primary outcome, because movement-evoked pain during the postoperative period is more severe, more frequent, and exerts a greater influence on postoperative functional rehabilitation compared with pain at rest [22]. Secondary outcomes included pain severity scores at rest (3 h, 6 h, 12 h, 24 h, 48 h, 72 h, 1w, 3w, and 3 m following surgery), morphine consumption (24 h, 72 h, and 1w following surgery), Harris Hip Score (HHS; 3w and 3 m following surgery), patient satisfaction at discharge, length of postoperative hospital stay and adverse events. Participants were requested to complete a 7-point satisfaction questionnaire before discharge [23]. Satisfaction levels ranged from being extremely satisfied to extremely dissatisfied. Adverse events were recorded until the last day on which duloxetine or the placebo was administered.

All data management and statistical analyses were conducted using IBM, SPSS version 22.0 software. Whereas the independent t-test was used to analyze differences between continuous variables, such as body mass index (BMI) and age, the chi-square test or Fisher’s exact test was used to analyze categorical variables. The significance level was set at *p* < 0.05.

## Results

**Fig. 1:**
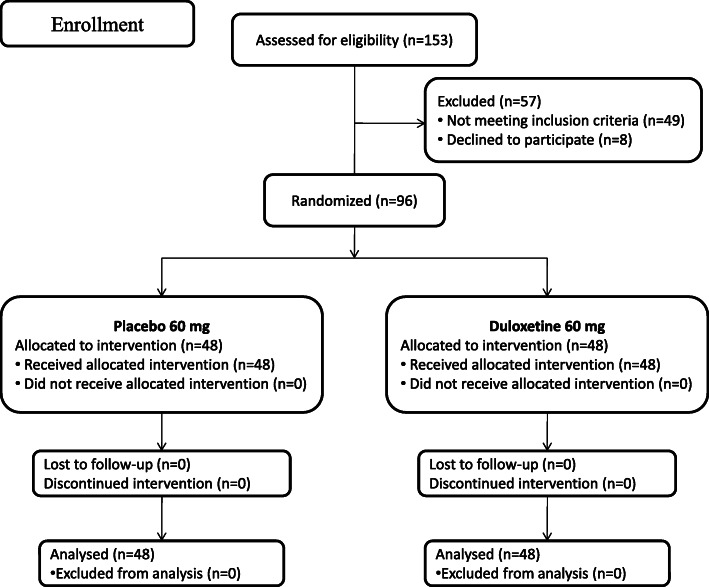
Schematic of the study design

A total of 153 patients with end-stage joint diseases were scheduled to undergo a primary unilateral THA procedure during the recruitment period (June 2020 to September 2020) at our department. Among these 153 patients, 49 did not satisfy the inclusion criteria and 8 declined to participate in the study. Thus, 96 eligible patients were included in the study. These patients were randomly assigned to either the duloxetine group or the placebo group. Specific information regarding patient flow is plotted (Fig. 1). There were no significant differences in the preoperative demographics and characteristics of the study participants between these groups (Table 1; *p* > 0.05).

**Table 1:** Preoperative demographics and characteristics

	Duloxetine (*n* = 48)	Placebo (*n* = 48)	*P* value
Demographic data			
Age^a^ (yr)	52.7 ± 12.0	50.2 + 13.2	0.333
Male sex^b^ (no.[%] of patients)	22 (46%)	24 (50%)	0.683
BMI^a^ (kg/m^2^)	24.0 ± 2.9	23.9 ± 3.4	0.890
ASA status^b^ (no.[%] of patients)			
I	8 (17%)	4 (8%)	
II	28 (58%)	32 (67%)	
III	12 (25%)	12 (25%)	
Surgical site (right/left)	27/21	24/24	
Diagnosis^b^ (no.of patients)			
Osteonecrosis of femoral head	16 (33%)	17 (35%)	
Primary osteoarthritis	15 (31%)	13 (27%)	
Developmental dysplasia of the hip	9 (19%)	10 (21%)	
Others	8 (17%)	8 (17%)	
Preoperative parameters^a^			
hip function			
Flexion (°)	91.7 ± 11.8	90.2 ± 15.8	0.610
Abduction (°)	21.5 ± 7.3	21.6 ± 8.4	0.948
Harris Hip Score (points)	42.1 ± 9.2	41.2 ± 10.6	0.652
BPI-pain severity score			
Average	5.4 ± 1.3	5.3 ± 1.1	0.666
Worst	7.6 ± 1.4	7.2 ± 1.7	0.128
Least	2.7 ± 1.2	2.7 ± 1.1	0.861
Current	5.4 ± 1.6	5.1 ± 1.2	0.243
BPI-interference score			
General activity	6.5 ± 2.0	6.5 ± 2.3	0.925
Walking	7.3 ± 1.9	7.4 ± 2.5	0.853
Work	6.5 ± 2.1	7.3 ± 2.3	0.082
Sleep	5.0 ± 2.1	4.5 ± 2.3	0.264
Relations with others	5.0 ± 2.3	4.7 ± 1.6	0.408
Enjoyment of life	4.8 ± 1.5	4.5 ± 1.6	0.430
Mood	5.2 ± 2.0	5.5 ± 2.4	0.512
HAMD	3.3 ± 1.3	3.0 ± 1.4	0.246
HAMA	3.4 ± 1.4	3.2 ± 1.4	0.425

*BPI* Brief Pain Inventory

^a^Data are given as the mean ± standard deviation

^b^Data are given as the number (percentage) of patients

The pain severity scores upon movement within the 3 postoperative weeks in patients in the duloxetine group were significantly lower compared to those in the placebo group (Table 2; *p* < 0.05). However, none of the between-group differences exceeds the MCID, and there was no significant difference between the pain severity scores upon movement in patients of the duloxetine and placebo groups after 3 months of surgery.

**Table 2:** Primary outcomes regarding VAS scores upon the movement

Time after surgery	Duloxetine* (*n* = 48)	Placebo* (*n* = 48)	*P* value†
3 h	6.0 ± 0.9	6.5 ± 1.2	**0.029**
6 h	5.9 ± 0.8	6.4 ± 1.0	**0.016**
12 h	5.4 ± 0.9	5.9 ± 1.0	**0.010**
24 h	5.0 ± 1.0	5.5 ± 1.2	**0.033**
48 h	4.4 ± 1.0	4.8 ± 1.2	**0.041**
72 h	3.9 ± 0.9	4.5 ± 1.1	**0.004**
1w	2.8 ± 1.0	3.5 ± 1.1	**< 0.001**
3w	1.9 ± 1.0	2.5 ± 1.1	**0.007**
3 m	1.6 ± 1.0	1.8 ± 1.0	0.469

*The values are given as the mean and standard deviation. †*P* values are calculated by independent t-test. *P* values indicating a significant difference among groups are in bold

Similar results were obtained for resting pain, wherein the post-operative movement-evoked pain level in patients of the duloxetine group was significantly lower than those in the placebo group until postoperative week 3 (Table 3; *p* < 0.05). There were no significant differences between the VAS scores for resting pain of the 2 groups after 3 months of surgery. Postoperative morphine consumption and HSS until 24 h, 72 h, and 1w after surgery are shown in Table 4. Patients in the duloxetine group required significantly less morphine compared to those in the placebo group (p < 0.05). There was no significant difference in HSS between the duloxetine group and the placebo group at either 21 d or 3 months after surgery. In terms of the satisfaction level at discharge, 45 patients in the duloxetine group expressed satisfaction (defined as extremely, very, or somewhat satisfied) with the treatment, compared to 36 patients in the placebo group (94% versus 75%, *p* = 0.011). No significant difference was observed between the lengths of postoperative hospital stay between the two groups. There was no significant difference in the adverse event between groups (Table 5; *p* > 0.05).

**Table 3:** Secondary outcomes regarding VAS scores for resting pain

Time after surgery	Duloxetine* (*n* = 48)	Placebo* (*n* = 48)	*P* value†
3 h	4.1 ± 1.0	4.5 ± 1.1	**0.026**
6 h	4.0 ± 1.0	4.4 ± 1.0	**0.032**
12 h	3.3 ± 1.0	3.8 ± 1.1	**0.044**
24 h	3.0 ± 1.1	3.5 ± 1.1	**0.046**
48 h	2.3 ± 1.0	2.8 ± 1.0	**0.019**
72 h	1.8 ± 0.9	2.2 ± 1.0	**0.023**
1w	1.0 ± 0.8	1.3 ± 0.8	**0.040**
3w	0.8 ± 0.7	1.1 ± 0.7	**0.043**
3 m	0.6 ± 0.5	0.7 ± 0.6	0.851

*The values are given as the mean and standard deviation. †P values are calculated by independent t-test. *P* values indicating a significant difference among groups are in bold

**Table 4:** Secondary outcomes regarding morphine consumption and HHS

	Duloxetine* (*n* = 48)	Placebo* (*n* = 48)	*P* value†
Morphine Consumption			
PO 24 h	11.0 ± 4.9	14.2 ± 5.9	**0.006**
PO 72 h	16.8 ± 6.1	20.4 ± 9.8	**0.032**
PO 1w	18.7 ± 7.3	23.3 ± 13.6	**0.039**
HHS			
PO 3w	75.4 ± 5,5	73.7 ± 6.1	0.156
PO 3 m	87.2 ± 4.7	87.8 ± 4.4	0.517

*PO* postoperative. *The values are given as the mean and standard deviation. †*P* values are calculated by independent t-test. *P* values indicating a significant difference among groups are in bold

**Table 5:** Secondary outcomes regarding satisfaction level, length of PO hospital stays and adverse events

	Duloxetine (*n* = 48)	Placebo (*n* = 48)	*P* value
Satisfaction level† (no.[%] of patients)			
Extremely satisfied	19 (40%)	11 (23%)	
Very satisfied	21 (44%)	17 (35%)	
Somewhat satisfied	5 (10%)	8 (17%)	
Neither satisfied nor dissatisfied	2 (4%)	6 (13%)	
Somewhat dissatisfied	1 (2%)	4 (8%)	
Very dissatisfied	0	2 (4%)	
Extremely dissatisfied	0	0	
length of PO hospital stays^a^ (h)	63.4 ± 17.6	70.7 ± 23.7	0.089
Adverse Events† (no.[%] of patients)			
Nausea and vomiting	8 (17%)	7 (15%)	0.779
Dry mouth	4 (8%)	2 (4%)	0.677
Insomnia	5 (10%)	6 (13%)	0.749
Somnolence	6 (13%)	3 (6%)	0.486
Constipation	7 (15%)	9 (19%)	0.584
Dizziness	3 (6%)	4 (8%)	1.000
Fatigue	5 (10%)	7 (15%)	0.537

^a^Data are given as the mean ± standard deviation. †Data are given as the number (percentage) of patients

## Discussion

In this trial, perioperative daily oral administration of 60 mg duloxetine from 2 d 

pre-operation to 14 d after surgery resulted in the lowering of postoperative movement-evoked pain and resting pain as well as in morphine requirements, while the pain differences between two groups are below the MCID. Moreover, patients expressed an increased satisfaction level at discharge from the hospital. Duloxetine did not appear to increase the incidence of adverse events.

The use of oral duloxetine in arthroplasty has been studied previously. Some of these studies have demonstrated its efficacy in reducing postoperative pain and opioid consumption [12, 14, 15]. As far as we know, all studies that have been conducted to determine the efficacy of duloxetine in alleviating residual pain following total joint replacement have focused on TKA and not on THA. Thus, the effect of orally administered duloxetine on THA remains unclear. Therefore, in an attempt to resolve the aforementioned issue, we conducted a randomized, controlled study to validate the effect of duloxetine on THA.

Pain, which follows arthroplasty, is a serious complication that always confounds orthopedists. Most recent literature indicates that long-lasting, intense, harmful pain stimuli induced by chronic joint diseases may trigger peripheral nociceptors and upregulate the excitability as well as synaptic efficacy of neurons in the central nociceptive pathways, leading to central sensitization. Central sensitization manifests as pain hypersensitivity and further triggers severe postoperative pain following arthroplasty [12, 13, 24–26]. Because serotonin signaling is involved in pain processing [27], the efficacy of SNRIs, including duloxetine, in resolving postoperative pain has been investigated [15].

In this study, we found that perioperative administration of duloxetine effectively relieved movement-evoked pain and resting pain 3 weeks post-surgery. These results are partially substantiated by a previously conducted randomized controlled trial, in which 80 patients scheduled for TKA, who were treated with duloxetine, achieved a better analgesic effect during the postoperative period from 2 to 12 weeks [12]. Except for the intrinsic difference between THA and TKA, the reason for the difference between the postoperative periods required to relieve pain may be partly attributed to the differences in the duration of the oral administration of duloxetine as well as its dosage. In our study, patients in the duloxetine group were administered duloxetine starting 2 d pre-surgery to 14 d post-surgery, as opposed to a previous study, which followed a protocol of administering 30 mg of duloxetine orally on the night before surgery and 30 mg per day for 6 weeks post-surgery. The Cochrane database review and other published literature have indicated that 60 mg of duloxetine administered daily was effective in treating painful neuropathy or chronic pain, whereas daily doses lower than 60 mg were ineffective [14, 15, 28]. Clinical pharmacokinetic studies reveal that duloxetine achieves maximum plasma concentration approximately 6 h after dosing and that its biological half-life is approximately 10–12 h [29]. Therefore, the protocol used for the perioperative administering of duloxetine in our study is believed to be appropriate and adequate. MCID is an important concept to put in perspective statistically significant results that may not be clinically relevant [30]. According to previous studies, we used an MCID for the VAS score of 2 in our study, while there were also other studies that determined MCID as other values [12, 18, 19]. To reduce the occurrence of side effects or complications, an analgesia protocol should preferably be multimodal [31]. When using a multimodal analgesia protocol, it is raising the bar very high to identify the performance of individual intervention, therefore, the findings which did not reach the MCID should have a role in a multimodal pain control protocol [31–33]. In a prospective, randomized controlled study comparing local infiltration anesthetic and control, authors found that the local analgesia group had a significantly lower mean VAS score for pain during exercise than did the control group (4.7 vs 6.6) on the first day after surgery (*p* = 0.008), and the difference for the VAS score of 1.9 offered improved pain control [31].

The findings of our study indicated that duloxetine reduced morphine consumption within 1 w post-THA. Two previous randomized controlled trials that enrolled 106 and 50 participants, respectively, also showed similar results [14, 15]. In one of the former studies, a daily oral dose of 60 mg duloxetine was administered to patients approximately 30 min prior to them being transferred to the operating room. The treatment was continued until 2 weeks after surgery and was found to significantly reduce the total opioid requirements for over a period of 3 months [15]. The other study revealed that two oral doses of 60 mg duloxetine, administered 2 h before surgery and on the first day after surgery, reduced morphine consumption in the first 48 h post-TKA [14]. The inconsistencies observed during the postoperative periods that warranted the reduction of opioid requirements in these two studies may be owing to the differences in assessment times as well as the dosage of duloxetine administered, which in turn are associated with the half-life of duloxetine.

The decision to use 60 mg of duloxetine as the daily dose in our study was based on the reports of previous studies [14, 15, 28]. Adequate quality-based evidence indicates that a daily dose of 60 mg of duloxetine is indeed the appropriate dosage [28]. Lower daily doses of duloxetine are not efficacious in alleviating pain; moreover, higher daily doses do not improve its efficacy and may even result in adverse events [28]. Our results also demonstrated that duloxetine did not increase the incidence of adverse events at a daily dose of 60 mg.

Our study had a few limitations. First, the 3-month follow-up time may obscure the long-term safety of duloxetine. However, the biological half-life of duloxetine is approximately 10–12 h, and maximum plasma concentration is attained approximately 6 h after dosing [29]. Patients with impaired liver and renal function were excluded during screening; therefore, the 3-month follow-up period was adequate for observing and treating adverse events. A second limitation was that the trial was performed at a single center, which may reduce generalizability. Nevertheless, generalizability is also influenced by different factors such as centers and individuals, as well as by inclusion and exclusion criteria. Lastly, the initial sample size estimation was based on our primary outcome, implying that this sample size may not be appropriate for determining a significant difference in terms of all relevant outcomes.

## Conclusions

In conclusion, the perioperative daily administration of 60 mg duloxetine to patients undergoing primary unilateral total hip arthroplasty alleviated movement-evoked pain 3 weeks post-surgery, although it did not result in a clinically meaning reduction in postoperative pain. Duloxetine also alleviated resting pain within 3 postoperative weeks, reduced morphine requirements following surgery, and improved satisfaction levels at discharge, without increasing the incidence of adverse events. Considering the positive results of the current study, it may be concluded that duloxetine shows potential as a novel therapeutic agent and could likely be used to optimize multimodal pain management protocols in total hip arthroplasty.

## Data Availability

The datasets used and/or analysed during the current study are available from the corresponding author on reasonable request.

## Abbreviation

THA: Total hip arthroplasty
SNRI: Selective serotonin and norepinephrine reuptake inhibitor
FDA: Food and Drug Administration
CS: Central sensitization
TKA: Total knee arthroplasty
ASA: American Society of Anesthesiologists
HAMD: Hamilton Depression Scale
HAMA: Hamilton Anxiety Scale
VAS: Visual analog scale
MCID: Minimum clinically important difference
HHS: Harris Hip Score
BMI: Body mass index
